# Occlusion of Bilobulated Left Atrial Appendage Using the Dual-Watchman Technique: A Long-Term Follow-Up Study

**DOI:** 10.3389/fcvm.2022.854475

**Published:** 2022-03-22

**Authors:** Tao Chen, Qing-song Wang, Ge Liu, Xu Lu, Ting-ting Song, Ming-yuan Shi, Hang Zhu, Yang Mu, Jun Guo, Yun-dai Chen

**Affiliations:** Senior Department of Cardiology, The Sixth Medical Center of People's Liberation Army (PLA) General Hospital, Beijing, China

**Keywords:** atrial fibrillation, left atrial appendage, occlusion, bilobulated LAA, LAAO

## Abstract

**Background:**

Percutaneous left atrial appendage (LAA) occlusion has been considered an efficient alternative to oral anticoagulation to prevent embolic events in patients with non-valvular atrial fibrillation (NVAF). Due to the complexities and heterogeneous anatomy of the LAA structure, the single-device approach may not always fit a large bilobulated LAA. This study aimed to evaluate the feasibility and safety of one-stop dual Watchman implantation for patients with bilobulated LAA.

**Methods:**

Included in the analysis were patients who underwent complete LAA closure with dual Watchman devices between December 2015 and December 2021. The anatomic morphology, procedure characteristics, procedure safety, and procedural complications were analyzed. Cardiac CT or transesophageal ultrasound was obtained at 7 days, 6 months, 1 year, and 2 years post-operatively to evaluate the effect of occlusion.

**Results:**

Among the 330 patients who underwent LAA occlusion during the study period, 7 (2.1%) patients were occluded with one-stop implantation of the double Watchman strategy. Successful occlusion was achieved in all patients. One patient had the double-access sheath strategy for implantation, and 6 patients had only a single-access sheath strategy for implantation. Pericardial effusion occurred in one case during the 7-day perioperative period. There was no device embolization, thrombosis, or obvious peridevice leakage (≥l mm) during the 2-year follow-up, with the exception of two cases with 2 mm of incomplete LAA sealing.

**Conclusion:**

The one-stop implantation of a dual Watchman is feasible and safe and might provide a strategy to occlude a large bilobulated LAA when incomplete closure is inevitable with a single device.

## Introduction

Atrial fibrillation (AF) is one of the most prevalent arrhythmias and most common causes of stroke and systemic embolism (1, 2). With the increasing implantation success rate and decreasing major adverse event rate, left atrial appendage occlusion (LAAO) has been recognized as an efficient treatment to prevent stroke in patients with non-valvular atrial fibrillation (NVAF) who cannot tolerate oral anticoagulation (3, 4). However, because of the complexities and heterogeneous anatomy of the left atrial appendage (LAA) structure, the single-device approach may not always fit the large bilobulated LAA (5, 6). Simultaneous and sequential implantation of dual devices has been reported for bilobulated LAA when an incomplete closure is inevitable with a single device, but experience with this strategy remains limited (7–10). We describe the assessment and closure strategy that was used in 6 patients with one-stop dual Watchman implantation for patients with bilobulated LAA.

## Methods

### Study Design

This is a retrospective, observational clinical study.

### Study Population

Among 330 patients who underwent successful LAAO in the Chinese People's Liberation Army General Hospital between December 2015 and December 2021, 7 patients with complex LAA anatomy had their LAA successfully closed with dual Watchman devices using the one-stop implantation technology. Procedural success was defined as a successful LAAO device implantation with adequate seal (<5 mm peridevice leak assessed using color flow Doppler). The demographical and clinical characteristics were recorded, including age, sex, congestive heart failure, hypertension, age ≥75 years, diabetes mellitus, stroke or transient ischemic attack (TIA), vascular disease, age 65–74 years, sex category (CHA2DS2-VASc) score, hypertension, abnormal liver/renal function, stroke history, bleeding history or predisposition, labile INR, elderly, drug/alcohol usage (HAS-BLED) score, and the anatomic characteristics of LAA. This study was approved by the Ethics Committee of the General Hospital of the Chinese People's Liberation Army. All patients signed informed consent form prior to the procedure.

### Imaging Data

**Figure 1:**
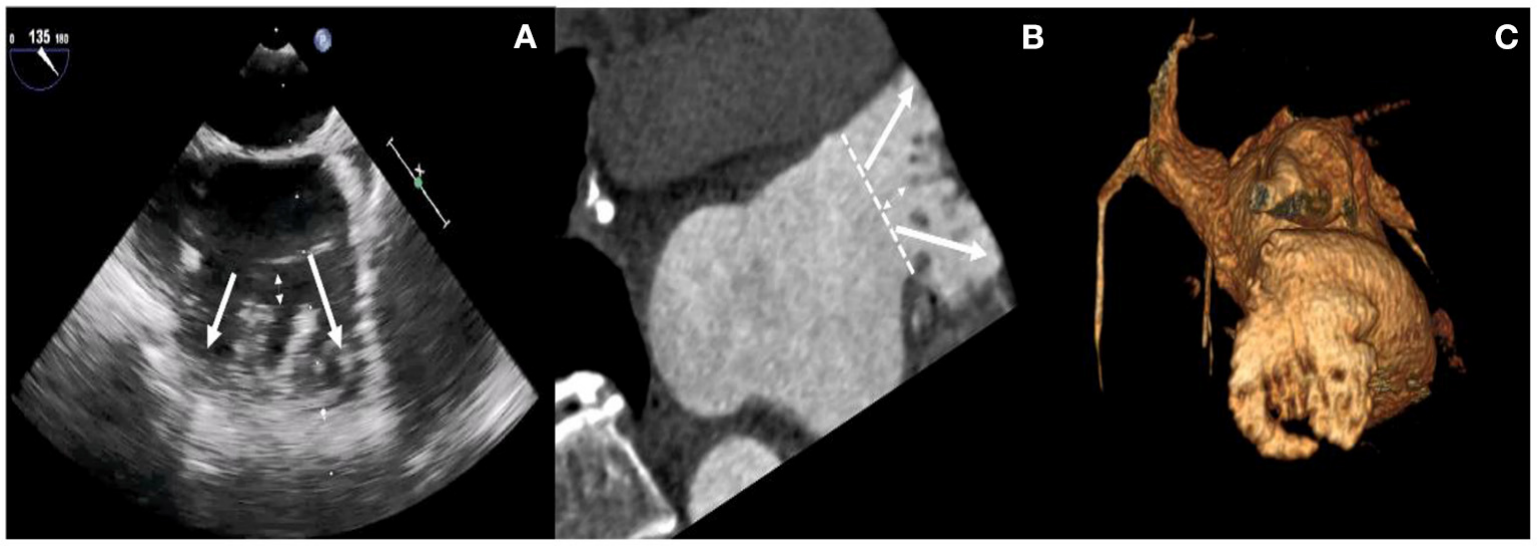
Higher degrees of 2D TEE and CCTA images. TEE views at 120° **(A)** reveal the presence of two main lobes with short common distances to the common ostium, a great diameter of ostium, and pectinate muscles that separated the LAA into multiple lobes (white arrows). Multiplanar reconstruction **(B)** and volume-rendering computed tomography (CT) image **(C)** provide an overview of the anatomy of LAA lobes and pectinate muscles.

Before the intervention, two-dimensional (2D) transesophageal echocardiography (TEE) or cardiac computerized tomography angiography (CCTA) was performed to exclude thrombi in the LAA. The size and morphology of the LAA were assessed by 2D TEE or CCTA. As shown in Figure 1, higher degrees of 2D TEE and CCTA showed a double-lobed LAA with short common distances to the common ostium, a great diameter of the ostium, and pectinate muscles that separated the LAA into two main lobes (Panel A–C).

### Implanting Strategy

**Figure 2:**
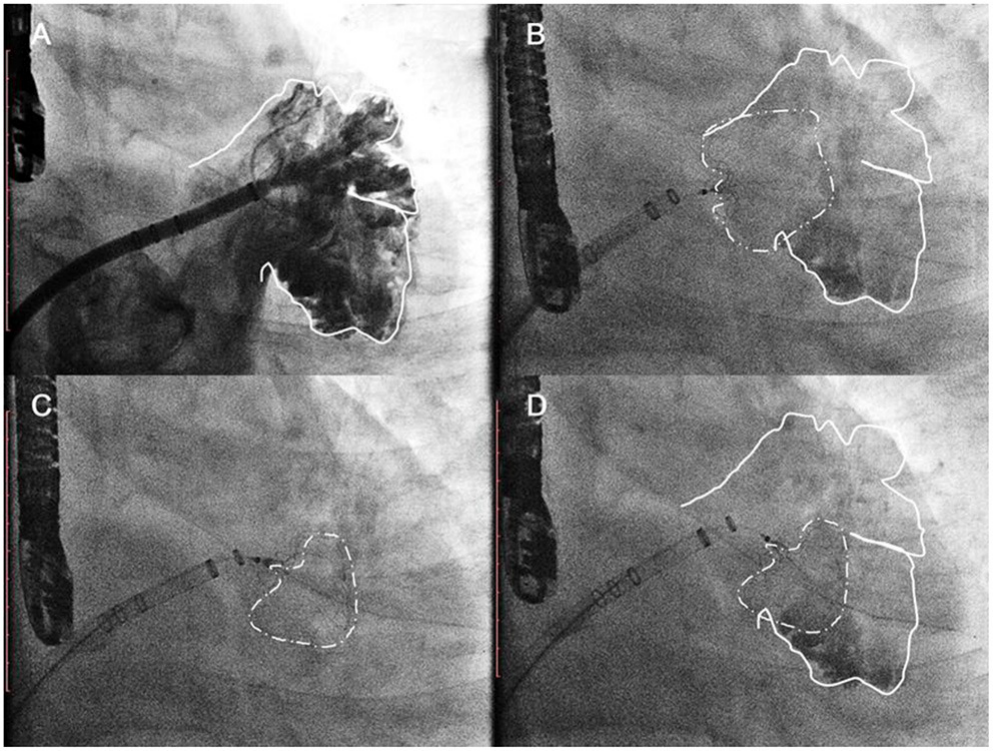
The common implantation strategy sequence. Step 1 **(A)** initial angiographic injection at right anterior oblique (RAO) 30°Caudal 20; Step 2 **(B)** a 33-mm Watchman device was initially chosen as the first attempt. Acquiring detail of the LAA anatomy through the TEE/CTA images was necessary before the dual-device strategy was selected. Step 3 **(C)** In the first implantation step, we chose an appropriately sized device to close the more challenging lobe of the LAA. Step 3 **(D)** The tug test and TEE were used to evaluate the PASS criteria with the first device.

The procedures were performed *via* femoral access and were guided by both TEE and angiography. A mid-transseptal puncture and a posterior transseptal puncture were performed. After the transseptal puncture, heparin was given to keep ACT above 250 s. Once selective angiograms were performed (Figure 2, Step 1), TEE and angiographic measurements were used to choose the size of the device. A 33-mm Watchman device was initially chosen for every case. However, if the LAA could not be completely sealed with a shoulder hanging outside the LAA (Figure 2, Step 2), the one-stop implantation of double Watchman was chosen for these patients. In the first-implantation step, we chose an appropriately sized device to close the more challenging lobe of the LAA (Figure 2, Step 3). The tug test and TEE were used to evaluate the Position, Anchor, Size, and Seal (PASS) criteria with the first device (Figure 2, Step 4).

#### Double-Sheath Strategy

**Figure 3:**
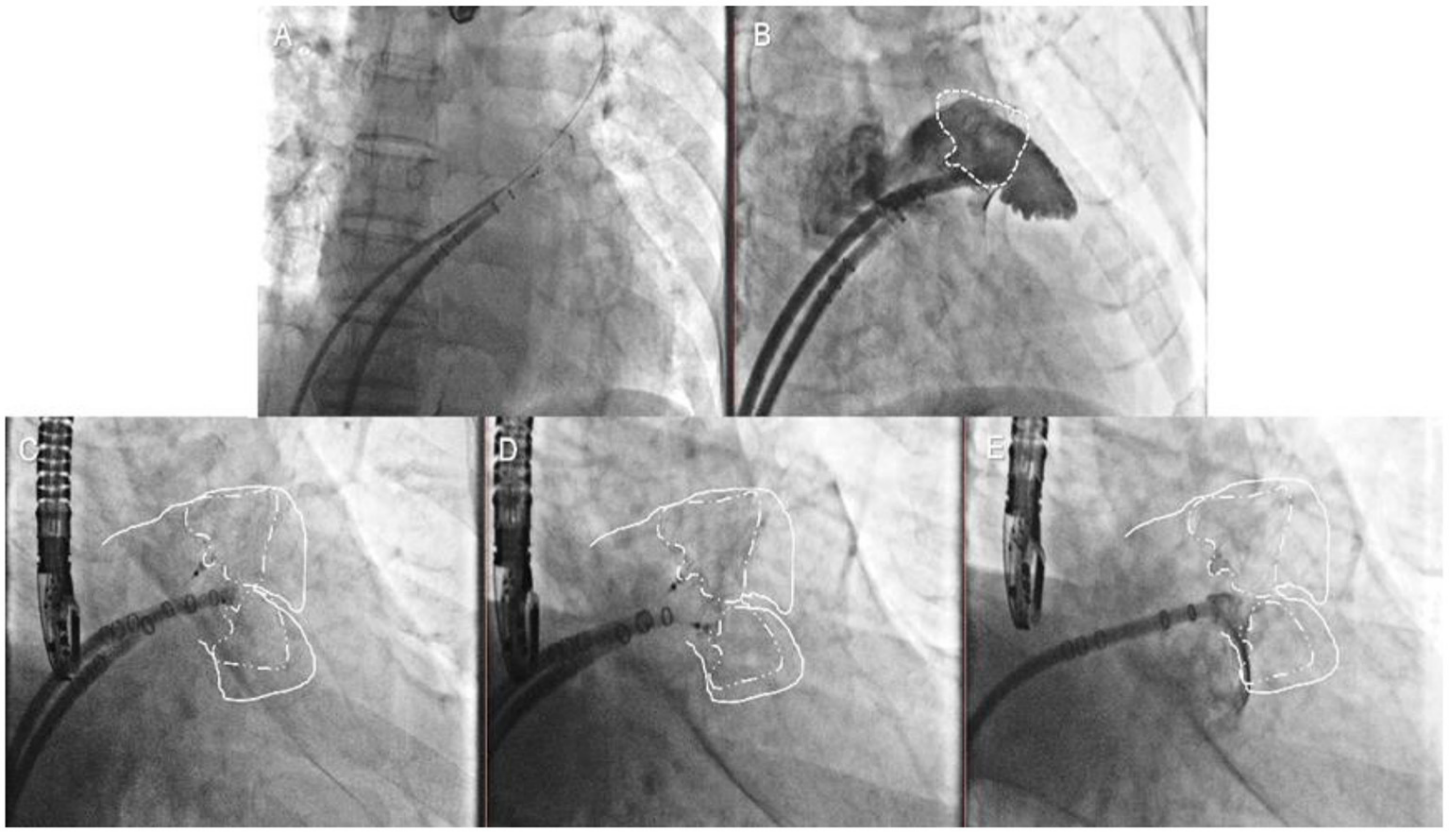
Double-sheath strategy. Step 5 **(A)** the second transseptal puncture *via* the same femoral site; Step 6 **(B)** the selective angiography of the second lobe was made; Step 7 **(C)** the second Watchman device was chosen according to the uncovered lobe; Step 8 **(D)** the tug test was performed on the two devices simultaneously; Step 9 **(E)** the two devices were released after the PASS criteria had been met with both devices.

In the first case, the second transseptal puncture was performed *via* the same femoral site (Figure 3, Step 5); the pigtail catheter with the second access sheath was delivered carefully to the uncovered lobe parallel to the first access sheath, and selective angiography was performed (Figure 3, Step 6). The second Watchman device was chosen according to the uncovered lobe and was carefully placed next to the first device (Figure 3, Step 7). The tug-test was performed on the two devices by pulling the parallel delivery system simultaneously (Figure 3, Step 8). The two devices were released after the PASS criteria had been met with both devices (Figure 3, Step 9).

#### Single-Sheath Strategy

**Figure 4:**
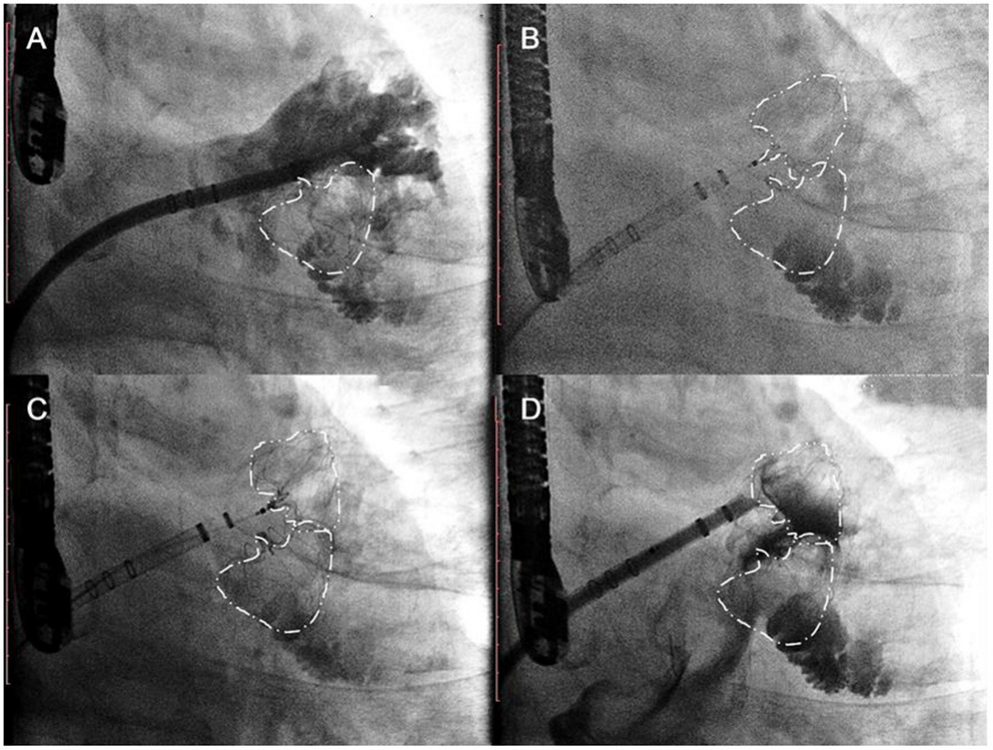
Single-sheath strategy. Step 5 **(A)** selective angiography of the second lobe was performed after the first device was released; Step 6 **(B)** the second Watchman device was chosen according to the uncovered lobe; Step 7 **(C)** the second tug test was performed carefully with the second devices; Step 8 **(D)** the second device was released after the PASS criteria had been met with both devices.

In the other cases, the first occluder was completely released after the PASS criteria had been met. The pigtail catheter with the access sheath was delivered carefully to the uncovered lobe, and selective angiography was performed (Figure 4, Step 5). The second-implantation step was similar to the first one but performed with more caution (Figure 4, Step 6). The second tug-test was performed with the second device (Figure 4, Step 7). The second device was released after the PASS criteria evaluation (Figure 4, Step 8).

### Medical Treatment and Follow-Up

After the implantation of the dual devices, all patients were treated with antithrombotic therapy for at least 45 days until there was no thrombus or obvious leakage (≥5 mm) around the device, which is confirmed by TEE or CTA follow-up at 6 weeks. Then, dual antiplatelet therapy (DAPT) with aspirin (100 mg/day) and clopidogrel (75 mg/day) was given until the second follow-up at 6 months. If thrombus or obvious leakage (≥5 mm) was excluded, aspirin was stopped and 75 mg/day clopidogrel was continued long term. TEE or CCTA was obtained at 7 days, 6 months, 1 year, and 2 years post-operatively to evaluate the effect of occlusion. Major adverse events during follow-up included device-related thrombosis, new renal insufficiency, serious pericardial effusion, device embolism, incomplete LAA sealing (enal), stroke, transient ischemic attack, systemic embolism, major bleeding, minor bleeding, device-related death, and all-cause death.

### Statistical Analysis

Categorical variables are given as frequencies (percentages). Normally distributed continuous variables are shown as the mean ± SD. Non-parametric continuous variables are listed as medians with interquartile ranges (IQRs). Statistical analyses were performed using the SPSS software (v 22.0; SPSS, Inc., Chicago, IL).

## Results

Among the 330 patients who underwent LAAO during the study period, 7 (2.1%) patients were occluded with dual watchman devices.

### Baseline Characteristics

Patient baseline and procedural characteristics are shown in Table 1. There were 6 men and 1 woman, and the mean age was 71.0 ± 6.9 years (range 57–79 years). The mean CHA2DS2-VASc score was 5.3 ± 1.3 points, and the mean HAS-BLED score was 4.0 ± 1.2 points. All the patients had a history of stroke. In terms of the rhythm status, 6 patients had persistent AF. The size and morphology of the LAA were assessed by 2D TEE or CCTA. All the patients had complex LAA anatomy with large cauliflowers. Each LAA had a large common ostium and 2 large main lobes, and each lobe could be occluded. The mean largest LAA size assessed using CCTA was 31.3 ± 1.8 mm, and the mean largest LAA size assessed using 2D TEE was 30.9 ± 2.5 mm. The appropriate technique was generally chosen according to the size and features of the LAA. One patient used the double-sheath strategy, and all others were occluded with only a single-access sheath. Successful implantation was achieved in all patients.

**Table 1:** Baseline and clinical characteristics of patients.

**Patient**	**No. 1**	**No. 2**	**No. 3**	**No. 4**	**No. 5**	**No. 6**	**No. 7**
Age	70	78	79	67	57	75	71
Gender	Male	Male	Male	Male	Male	Female	Male
CHA2DS2-VASc score	6	5	6	7	4	6	3
HAS-BLED score	4	3	5	6	4	4	2
LVEF (%)	52	55	56	48	52	65	30
Persistent AF	Yes	Yes	No	Yes	Yes	Yes	Yes
CHF	No	No	No	Yes	No	No	Yes
CHD	Yes	Yes	Yes	Yes	Yes	Yes	No
Hypertension	Yes	No	Yes	Yes	Yes	Yes	No
DM	Yes	No	No	Yes	No	No	No
Stroke	Yes	Yes	Yes	Yes	Yes	Yes	Yes
Bleeding history	Yes	No	No	Yes	Yes	No	No
Largest LAA size by angiography (mm)	30	30	31	30	35	30	33
Largest LAA size by 2D TEE (mm)	29	28	33	29	35	29	33
Device size (mm)	24/33	24/27	24/27	30/33	24/27	24/30	24/27
No. of access sheath	2	1	1	1	1	1	1
Peridevice leakage (mm)	2	0	2	0	0	0	0
Pericardial effusion	No	No	No	No	No	Yes	No

*LVEF, left ventricular ejection fraction; CHF, congestive heart failure; CHD, coronary heart disease; DM, diabetes mellitus; LAA, left atrial appendage*.

### Major Adverse Events and Follow-Up

**Figure 5:**
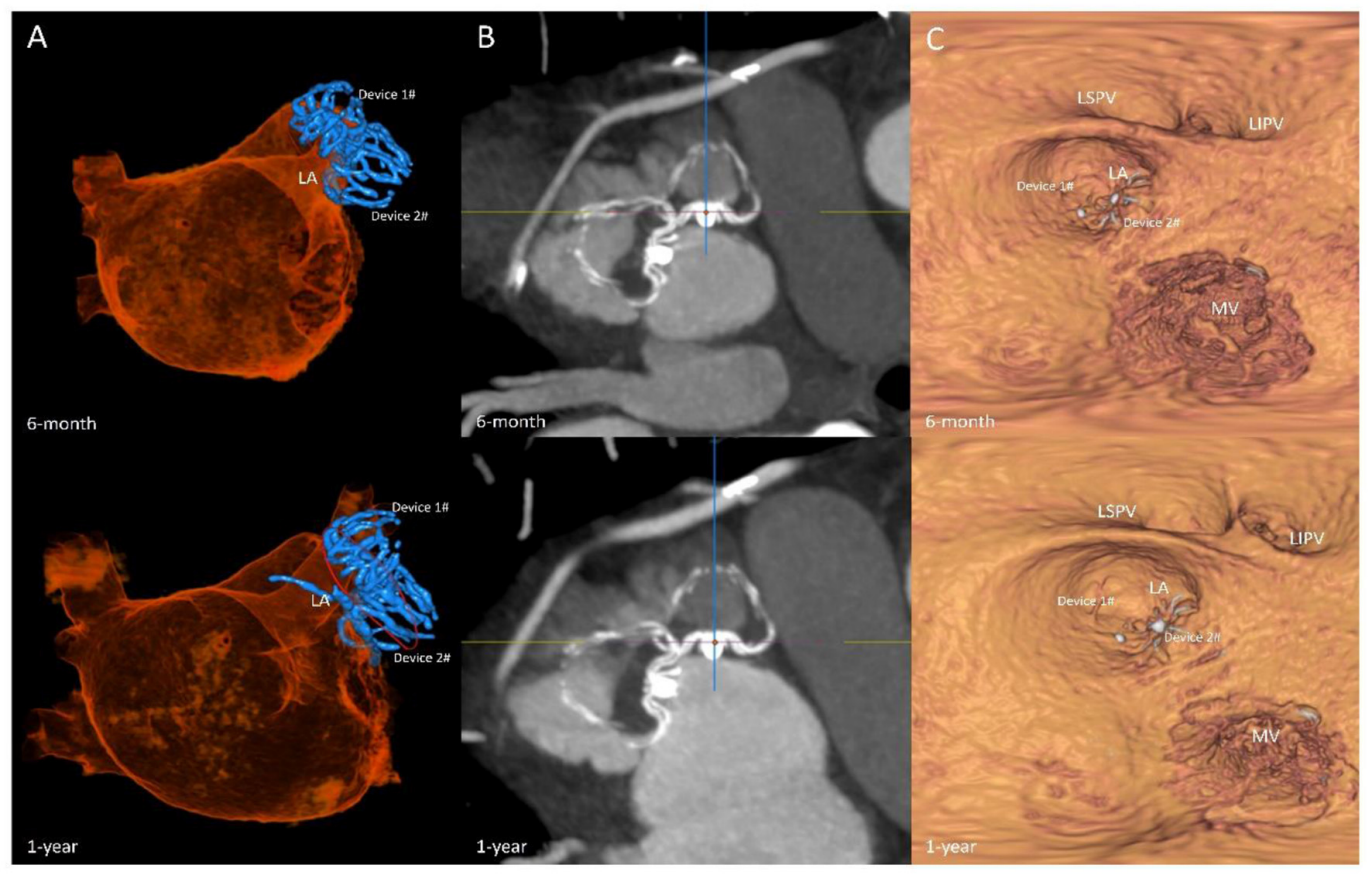
Follow-up imaging. Multiplanar reconstruction evaluating the anatomic position **(A)**; volume-rendering CT image evaluating the residual shunt after surgery **(B)**; multiplanar reconstruction evaluating the endothelialization of the occlusions **(C)**.

Among the 7 patients, 1 patient developed pericardial effusion during the 7-day perioperative period. The patient was discharged from the hospital after pericardiocentesis and drainage and adjustment of anticoagulants. TEE and CCTA were obtained at 7 days, 6 months, 1 year, and 2 years post-operatively to monitor device residual shunt, device-related thrombosis, and adverse events and to evaluate the effectiveness of occlusion. Follow-up results are shown in Table 2. There was no device embolization, thrombosis or obvious peridevice leakage (≥l mm) during the 2-year follow-up, with the exception of 2 patients with 2 mm of incomplete LAA sealing. The residual shunt occurred in the free links between the occluder and the rim of the defect or the occluder itself. Over time, endothelialization covered the surface of the packer and then reduced the residual shunt. The 6-month and 1-year follow-up images of one patient are shown in Figure 5.

**Table 2:** Major adverse events within 2-year follow-up after the procedures.

**Events Post-operative time**	**7 Days (No.1 to No.7)**	**6 Months (No.1 to No.7)**	**1 Year (No.1 to No.7)**	**2 Years (No.1 to No.7)**
Device related thrombosis, *n* (%)	0	0	0	0
Newly renal insufficiency, *n* (%)	0	0	0	0
Serious pericardial effusion, *n* (%)	1(16.7%)	0	0	0
Device embolism, *n* (%)	0	0	0	0
Incomplete LAA sealing				
<5 mm, *n* (%)	2 (33.3%)	2 (33.3%)	2 (33.3%)	2 (33.3%)
≥5 mm, *n* (%)	0	0	0	0
Stroke, *n* (%)	0	0	0	0
Transient ischemic attack, *n* (%)	0	0	0	0
Systemic embolism, *n* (%)	0	0	0	0
Major bleeding, *n* (%)	0	0	0	0
Minor bleeding, *n* (%)	0	0	0	0
Device related death, *n* (%)	0	0	0	0
All cause death, *n* (%)	0	0	0	0

## Discussion

The anatomy of the LAA is highly variable, and bilobulated LAA anatomy is a challenging situation for LAAO with the currently available devices (7, 11). It is a huge obstacle to close the LAA with a single Watchman device for an LAA that has complex characteristics, including cauliflower morphology, a maximum body diameter (>30 mm), two main lobes, or the crista of musculi pectinate. Under this circumstance, a strategy with dual devices has been used to occlude bi- or multilobar LAAs in previous registries (6–8). However, clinical answers are still lacking regarding anatomical considerations, device type and size, single approach or staged, and single-sheath or double-access sheath.

In this study, all the patients had their LAA successfully occluded with dual Watchman devices with a single-stage strategy. Six out of seven patients who underwent successful LAA occlusion were treated with the single-sheath strategy. Hence, a second transseptal puncture was not performed for these patients, which can reduce the procedure steps and minimize the risk of persistent iatrogenic atrial septal defects.

Incomplete sealing and residual peridevice leaks were reported in previous reports as well as in our cases, and they have been linked to a higher risk of cardioembolic events in patients undergoing surgical LAA closure. To date, late mechanical interaction and device-related thrombosis between the devices are unknown. Therefore, we do not recommend dual devices as the first choice. A careful review of the details of the LAA anatomy is very important before single-stage dual-device implantation. In our cases, a ridge-like pectinate muscle separated the LAA into two main lobes, and this anatomic structure warrants that the dual devices were implanted separately but not adjacently, reducing the possibility of interaction between devices. The other factor to consider is which lobe is to be occluded first. In our experience, the harder lobe should be closed as the first choice. Immediately after the first device was prereleased, the uncovered lobe was assessed by TEE. If the first device was not too close to the other lobe, the first device was completely released after the confirmation of exposure with angiography *via* the delivery system and TEE imaging.

Only the Watchman device is commercially available in our hospital. It is possible that the availability of other occluders, such as Watchman FLX or LAmbre, would limit the need for the two-device technique. The Watchman device was a parachute-shaped device without an additional proximal disk to seal the ostium. For these special LAAs, we were concerned about the reaction between the dual devices and long-term endothelialization. Compared with the first-generation Watchman device, the dual-disk device was non-inferior with respect to safety and effectiveness endpoints and superior with respect to LAA occlusion (12). According to the instructions, the largest size of Amulet is 32 mm. However, the mean largest LAA size assessed by CCTA was 31.3 ± 1.8 mm in our study, which suggests that Amulet might not be appropriate in these patients. Hence, we selected dual Watchman devices as the alternative choice.

To ensure the long-term efficacy of LAAO, TEE and CCTA are commonly used to monitor device residual shunts, device-related thrombosis, and adverse events to evaluate the effectiveness of occlusion (13, 14). In our follow-up study, TEE and CCTA were obtained at 7 days, 6 months, 1 year, and 2 years post-operatively to evaluate the effect of occlusion. Four patients underwent CCTA, and two underwent TEE. In patients who underwent complete LAA closure with dual devices, residual shunts were observed in 28.6% of patients (2 of the 7 patients) in our center. Fortunately, none of our 7 patients developed device-related thrombi.

Pericardial effusion is a serious complication after LAAO (15). In our study, one patient developed pericardial effusion 2 h after the procedure and received pericardiocentesis 5 ho after the procedure. However, the whole surgical procedure was uneventful. We did not perform many surgical procedures that may increase the risk of pericardial effusion, such as device retrieval during surgery. The occurrence of pericardial effusion may be related to the use of anticoagulants (16). We analyzed the possible causes of the complications. The patient was treated with warfarin before surgery for anticoagulation and received combined catheter ablation and LAAO. The intraprocedural mean ACT value was 450. The international normalized ratio (INR) measured after the operation was 3.5 due to inappropriate anticoagulation strategies. A high INR increases the risk of pericardial effusion, which could be considered a cause of unexplained recurrent pericardial effusion. The definite correlation of dual-device implantation in the incidence of pericardial effusion remains uncertain. The patient's condition was controlled by the third day after timely pericardiocentesis and drainage and adjustment of anticoagulants. The patient was discharged from the hospital after symptomatic treatment, and no complications occurred during follow-up.

## Limitations

This was a retrospective study with a small sample size. According to our LAA closure patient data, only seven cases matched these special complex characteristics. More cases and experiences are being collected. Double-device closure has been reported for bilobulated LAAs, but it still lacks clinical answers on anatomical considerations, device type, one-stop or staged, and single sheath or double sheath. Only the Watchman device is commercially available in the United States and parts of China. It is possible that the availability of other occluders would limit the need for the dual-device technique.

## Conclusion

In brief, we provided primary evidence on a single-access sheath strategy to deliver two Watchman devices to occlude the LAA with challenging anatomies in this study. According to our results, this single-sheath strategy is feasible and safe, which might reduce the number of procedural steps and minimize iatrogenic atrial septal damage. The long-term safety and efficacy of this technique remain to be assessed.

## Data Availability Statement

The original contributions presented in the study are included in the article/supplementary material, further inquiries can be directed to the corresponding author/s.

## Ethics Statement

The studies involving human participants were reviewed and approved by the Ethics Committee of the General Hospital of the Chinese People's Liberation Army. The patients/participants provided their written informed consent to participate in this study. Written informed consent was obtained from the individual(s) for the publication of any potentially identifiable images or data included in this article.

## Author Contributions

Y-dC and JG designed and supervised the study. TC and Q-sW collected the data and wrote the article. GL, T-tS, M-yS, HZ, and YM collected the data and assisted with writing of the article. XL was responsible for the statistical design of the study and for carrying out the statistical analysis. All authors contributed to the article and approved the submitted version.

## Funding

This study was supported by grants from the Beijing Nova Program (Z18100006218120 to TC), the Youth Innovation Promotion Association of the Chinese Academy of Sciences (17-JCJQ-QT-029 to TC), and the National Key R & D Program of China (2018YFCZ001200 to JG).

## Conflict of Interest

The authors declare that the research was conducted in the absence of any commercial or financial relationships that could be construed as a potential conflict of interest.

## Publisher's Note

All claims expressed in this article are solely those of the authors and do not necessarily represent those of their affiliated organizations, or those of the publisher, the editors and the reviewers. Any product that may be evaluated in this article, or claim that may be made by its manufacturer, is not guaranteed or endorsed by the publisher.
